# Hemostatic Dysfunction Is Increased in Patients with Hepatosplenic Schistosomiasis Mansoni and Advanced Periportal Fibrosis

**DOI:** 10.1371/journal.pntd.0002314

**Published:** 2013-07-18

**Authors:** Luiz Arthur Calheiros Leite, Adenor Almeida Pimenta Filho, Caíque Silveira Martins da Fonseca, Bianka Santana dos Santos, Rita de Cássia dos Santos Ferreira, Silvia Maria Lucena Montenegro, Edmundo Pessoa Lopes, Ana Lúcia Coutinho Domingues, James Stuart Owen, Vera Lúcia de Menezes Lima

**Affiliations:** 1 Departamento de Bioquímica, Centro de Ciências Biológicas, Universidade Federal de Pernambuco (UFPE), Recife, Brazil; 2 Departamento de Medicina Tropical, Centro de Ciências da Saúde, UFPE, Recife, Brazil; 3 Departamento de Imunologia, Centro de Pesquisa Aggeu Magalhães (CPqAM)/FIOCRUZ - PE, Fiocruz, Brazil; 4 Departamento de Medicina Clínica, Centro de Ciências da Saúde, Hospital das Clinicas, UFPE, Recife, Brazil; 5 Division of Medicine, University College London Medical School, Royal Free Campus, London, United Kingdom; Hospital Universitário, Brazil

## Abstract

**Background:**

Schistosomiasis mansoni is an endemic parasitic disease and a public health problem in Northeast Brazil. In some patients, hepatic abnormalities lead to periportal fibrosis and result in the most severe clinical form, hepatosplenic schistosomiasis. This study aimed to evaluate whether abnormal blood coagulation and liver function tests in patients with hepatosplenic schistosomiasis (n = 55) correlate with the severity of their periportal fibrosis.

**Methodology/Principal Findings:**

Blood samples were used for liver function tests, hemogram and prothrombin time (International Normalized Ratio, INR). The blood coagulation factors (II, VII, VIII, IX and X), protein C and antithrombin IIa (ATIIa), plasminogen activator inhibitor 1 (PAI-1) and D-dimer were measured by photometry or enzyme linked immunosorbent assay. Hyperfibrinolysis was defined on the basis of PAI-1 levels and a D-dimer concentration greater than a standard cut-off of 483 ng/mL. Standard liver function tests were all abnormal in the patient group compared to healthy controls (n = 29), including raised serum transaminases (p<0.001) and lower levels of albumin (p = 0.0156). Platelet counts were 50% lower in patients, while for coagulation factors there was a 40% increase in the INR (p<0.001) and reduced levels of Factor VII and protein C in patients compared to the controls (both p<0.001). Additionally, patients with more advanced fibrosis (n = 38) had lower levels of protein C compared to those with only central fibrosis (p = 0.0124). The concentration of plasma PAI-1 in patients was one-third that of the control group (p<0.001), and D-dimer levels 2.2 times higher (p<0.001) with 13 of the 55 patients having levels above the cut-off.

**Conclusion/Significance:**

This study confirms that hemostatic abnormalities are associated with reduced liver function and increased liver fibrosis. Of note was the finding that a quarter of patients with hepatosplenic schistosomiasis and advanced periportal fibrosis have hyperfibrinolysis, as judged by excessive levels of D-dimer, which may predispose them to gastrointestinal bleeding.

## Author Summary

Schistosomiasis is a parasitic disease that affects the liver and in the severe hepatosplenic form results in periportal fibrosis. This disease is a major public health problem in Northeast Brazil. Our study aim was to evaluate whether abnormal blood coagulation and liver function tests in patients with hepatosplenic schistosomiasis depended on the severity of their fibrosis and could be used to inform diagnosis and treatment. We verified, by analyzing blood samples and by abdominal ultrasound of 55 patients, that blood clotting abnormalities are associated with reduced functioning of the liver and with increased liver fibrosis. Our results additionally suggested that reduced levels of protein C in plasma are a good marker of liver fibrosis progression. Also of note was our finding that a quarter of patients with advanced fibrosis have hyperfibrinolysis, a severe blood clotting disorder which may increase their risk of gastrointestinal bleeding. Therefore, we recommend, for patients eligible for surgical procedures, that certain blood tests (D-dimer, prothrombin time and platelet count) be measured during the pre-surgical evaluation to better assess risk of bleeding.

## Introduction

Schistosomiasis is a chronic parasitic liver disease that constitutes a major public health problem in several parts of the world. There are more than 200 million people affected by schistosomiasis worldwide and 600 million people are at risk of infection [1]–[3]. The disease caused by *Schistosoma mansoni* is the most prevalent liver disease in the Northeast region of Brazil [4]. Around 5–7% of patients infected by *S. mansoni* progress to the most severe form, hepatosplenic. Many patients exhibit high morbidity and mortality associated with periportal fibrosis, portal hypertension and splenomegaly, which lead to frequent episodes of upper gastrointestinal bleeding [5].

Periportal fibrosis constitutes the pathognomonic lesion of the liver in hepatosplenic schistosomiasis [6]–[8]. This process results from massive deposition of collagen products in the periportal spaces and leads in turn to progressive occlusion of the portal vein, portal hypertension, splenomegaly, collateral venous circulation and bleeding of the upper gastrointestinal tract. GI bleeding episodes are one of the causes of hepatic dysfuction in schistosomiasis; areas of hepatic necrosis can occur due to hypotension and loss of blood, which in liver regeneration can distort the hepatic parenchyma. When patients bleed more than once, hepatic dysfunction and compromised hemostasis ensue [6].

A more extensive pattern of fibrosis reflects the prognosis and severity of the chronic hepatosplenic condition [6], although it is generally reported that liver function remain preserved. On the other hand, some studies have found reduced levels of blood coagulation proteins, which are synthesized by liver cells [9]–[11]. Classically, it is believed that liver cell function is preserved in hepatosplenic schistosomiasis and that the compromised hemostasis is due to a consumptive coagulopathy related to the enlarged liver and spleen. In a previous study, it was suggested that early liver dysfunction in schistosomiasis may contribute to the problems with hemostasis [9]. However, it remains unclear whether an advanced pattern of fibrosis is linked to an adverse effect on hemostasis and liver function. Here, our aim was to determine whether abnormal blood coagulation and liver function tests in patients with hepatosplenic schistosomiasis correlate with the severity of their periportal fibrosis.

## Materials and Methods

### Ethical statement

The study was conducted according to the Helsinki Declaration and was approved by the Human Research Ethics Committee of the Federal University of Pernambuco (Number 028/11), in Brazil. All patients and healthy subjects received an explanation about the scope of the study, such as objectives, procedures and potential risks, and signed an informed consent statement before inclusion in the study.

### Patients

Fifty-five patients diagnosed with hepatosplenic schistosomiasis, and previously treated with praziquantel (50 mg/Kg) at least 6 months before the present study, were the overall of those attending as outpatients between 2010 and 2012 at the Gastroenterology Department, Clinical Hospital of the Federal University of Pernambuco, Recife, Brazil. When first seen at the clinic all patients had hepatosplenomegaly and portal hypertension, but without ascites, jaundice, encephalopathy and/or pulmonary hypertension, and a history of contact with river water within municipalities located in “Zona da Mata”, an endemic area for schistosomiasis in Pernambuco State. Some had reported at least one episode of upper gastrointestinal bleeding.

The diagnosis of schistosomiasis was based on clinical history, physical examination and an abdominal ultrasonography which showed periportal fibrosis. Using the World Health Organization (Niamey Working Group, 2000) protocol [12] patients were classified as having peripheral fibrosis (Pattern C), central fibrosis (Pattern D), advanced fibrosis (Pattern E) or very advanced fibrosis (Pattern F) [12], [13]. Patients who presented with advanced or very advanced fibrosis were grouped together for data analyses (Pattern E+F).

Patients were excluded if they reported alcohol abuse (>60 g ethanol/day for men and >40 g/day for women) or had a history of splenectomy, hepatic cirrhosis, systemic diseases such as diabetes mellitus, acute or chronic hepatitis B or C, collagenosis, heart and blood diseases. Use of hepatotoxic drugs, acetylsalicylic acid, anticoagulant drugs, or receiving a blood transfusion were also criteria for exclusion if less than 90 days prior to data collection. The control group consisted of twenty nine healthy individuals from the same age range (18 to 65 years) and socioeconomic background, as evaluated by a standardized questionnaire that enabled family budget, education level and lifestyle to be matched with those of the patients.

### Sample collection and processing

Three venous blood samples were collected under aseptic conditions without stasis using vacuum tubes (Vacutainer; *Becton Dickinson*, USA). The first tube contained 0.106M trisodium citrate at a 1∶9 ratio to blood for coagulation tests. The second contained 0.562M EDTA-K3 and was used directly for platelet quantification, while the third blood collection tube was used for liver function tests. Tubes one and three were centrifuged for 10 min at 2000 *g* and the plasma and serum stored in 0.5 ml aliquots at −80°C until assayed.

### Parasitological diagnosis

Stool samples from control and patient groups were taken on two consecutive days and each tested twice by the Kato-Katz method. The mean egg counts are reported.

### Biochemical, hepatic and other tests

The routine liver function tests included aspartate and alanine aminotransferases (AST and ALT), alkaline phosphatase (ALP), γ-glutamyltransferase (γGT), bilirubin (Total, Direct and Indirect) and albumin and were measured by automated spectrophotometry (Cobas C501, Roche, Diamond Diagnostics, USA). Determinations of HBsAg, anti-HBc and anti-HCV were made by Chemiluminescence Microparticle Immuno Assay (CeMIA) using the ARCHITECT i2000 automatic light detector and test reagents (Abbott, North Chicago, USA) to exclude enrollment of patients with Hepatitis B or C. Abdominal ultrasound avoided inclusion of patients with hepatic cirrhosis and steatosis, and the anamnesis excluded patients with active use of alcohol.

### Platelet count and blood coagulation tests

Platelet counts (normal range 150–400×10^9^/L) were measured by electrical impedance using the Pentra-120 (ABX Diagnostics, São Paulo, SP, Brazil). Coagulation tests were performed with an automated photooptical coagulometer, (Trinity Biotech, Acton, USA) and included measuring prothrombin time (PT, expressed as the INR), partial thromboplastin time (PTT), thrombin test (TT) and fibrinogen.

Blood coagulation factors II, VII, VIII, IX, X were assayed using a Destiny Plus analyzer (Trinity Biotech, Acton, USA) and were based on correcting the long PT of factor deficient plasma by addition of test plasma diluted with clotting factor deficient plasma. Results were expressed as a percentage of activity for each factor. Protein C in test plasma was measured in the Destiny Plus analyzer with a specific snake venom protein C activator, thus inhibiting factor V and VIII in the added Protein C deficient plasma reagent and prolonging the subsequent PTT test, while antithrombin IIa was determined using saline buffer and specific reagents.

Antigenic assays to quantify tissue plasminogen activator (t-PA), plasminogen activator inhibitor −1 (PAI-1) and thrombin-activatable fibrinolysis inhibitor (TAFI) in plasma were measured by sandwich enzyme-linked immunosorbent assays ELISA (Asserachrom, Diagnostica Stago, France). Each sample was tested in duplicate and measured according to the supplier's instructions. D-dimer was assayed in an automated photo-optical coagulometer; values above 483 ng/mL were defined as hyperfibrinolysis [14].

### Statistical analysis

Unpaired Student's *t* test was used to compare differences between normally distributed variables of the hepatosplenic schistosomiasis patients (combined total) and control group, while the fibrosis pattern groups were compared by one-way analysis of variance (ANOVA) followed by Fisher's protected least significant difference (PLSD) test. Mann-Whitney and Kruskal-Wallis followed by Dunn's multiple comparison tests were used to compare differences among non-normally distributed variables. Variables were expressed as mean±Standard Error of the mean. P-values less than 0.05 were considered to be statistically significant. All statistical analyses were performed using Statview SAS Inc. (1998, NC, USA).

## Results

All patients presented with some degree of periportal fibrosis. The Niamey classification [12] revealed a predominance of advanced periportal fibrosis, Pattern E (n = 30; 54.6%), followed by central fibrosis, Pattern D (n = 17; 30.9%), while only 8 of the patients (14.5%) showed Pattern F, very advanced fibrosis. No patient was classified as having Pattern C (peripheral fibrosis). For easiness of data analysis, the two patterns of advanced periportal fibrosis were combined into a single group (E+F; n = 38; 69.1%).

As a total group, the patients with hepatosplenic schistosomiasis showed abnormal liver function tests compared to the healthy controls with significantly (p<0.05) increased levels of serum AST, ALT, γ-GT, ALP and total bilirubin, and a lower concentration of albumin (Table 1). These differences were also seen when the two patients groups (Patterns D and E+F) were compared separately with the controls, except for the albumin level in the Pattern D group which was not significantly reduced. No differences were noted when patients with central fibrosis (Pattern D) were compared to those with advanced periportal fibrosis (Pattern E+F) although the level of γ-GT was 64% higher in the latter group (p = 0.0501; Table 1).

**Table 1:** Liver function tests in hepatosplenic schistosomiasis patients with different patterns of periportal fibrosis.

Characteristics	Controls (C)	Hepatosplenic Schistosomiasis Patients			p-value			
		Overall	D fibrosis pattern	E+F fibrosis pattern	Overall vs. C	D vs. C	E+F vs. C	D vs E+F
Subjects (n)	29	55	17	38	-	-	-	
AST (U/L)	21.9±1.2	51.6±4.7	54.4±9.3	50.4±5.4	<0.0001	0.0003	0.0001	0.6276
ALT (U/L)	18.6±0.9	49.2±5.8	57.5±10.9	45.4±6.9	0.0003	0.0005	0.0027	0.2399
ALP (U/L)	65.2±3.4	170.7±23.5	162.3±21.4	174.4±32.8	0.0017	0.0287	0.0026	0.7716
γ-GT (U/L)	27.2±2.6	145.0±19.2	103.4±24.1	169.4±25.1	<0.0001	0.0313	<0.0001	0.0501
Albumin (g/dL)	4.30±0.07	3.98±0.08	4.03±0.12	3.96±0.11	0.0156	0.1189	0.0170	0.6834
Total bilirubin (g/dL)	0.65±0.05	1.23±0.12	1.18±0.16	1.26±0.16	0.0011	0.0239	0.0017	0.7345
Direct bilirubin (g/dL)	0.33±0.03	0.52±0.08	0.43±0.08	0.57±0.12	0.1050	0.5333	0.0667	0.3624
Indirect bilirubin (g/dL)	0.31±0.04	0.71±0.07	0.75±0.15	0.69±0.08	0.0002	0.0017	0.0008	0.6343

Values are expressed as mean±Standard Error (SE). Unpaired Student's *t* test or one-way ANOVA followed by Fisher's PLSD post test.

The combined total groups of patients all showed significant increases in the INR, PTT and TT values compared to the healthy controls, while the platelet count (×10^9^/L) was 50% lower (128±13 vs. 261±10; p<0.001) (Table 2). All blood coagulation factors (II, VII, VIII, IX, X and antithrombin IIa) were lower in the total patient group, and these significant differences were also seen when Pattern D and Pattern E+F were compared with the healthy controls as separate groups (Table 2). However, only protein C was significantly different between the two patient groups (74.5±5.1% for Pattern D vs. 61.6±3.3% for Pattern E+F; p = 0.0124).

**Table 2:** Coagulation parameters from hepatosplenic schistosomiasis patients with different patterns of periportal fibrosis.

Characteristics	Controls (C)	Hepatosplenic Schistosomiasis Patients			p-value			
		Overall	D fibrosis pattern	E+F fibrosis pattern	Overall vs. C	D vs. C	E+F vs. C	D vs. E+F
Subjects (n)	29	55	17	38	-	-	-	-
Platelets Count (×10^9^/L)	261.1±9.8	128.4±12.7	146.6±23.1	120.3±14.0	<0.0001	<0.0001	<0.0001	0.1112
INR	1.01±0.02	1.44±0.06	1.38±0.09	1.47±0.07	<0.0001	0.0028	<0.0001	0.4828
TT (Seconds)	11.9±0.2	13.8±0.2	13.56±0.4	13.85±0.3	<0.0001	0.0001	<0.0001	0.4770
PTT (Seconds)	31.1±0.6	37.9±1.5	36.4±1.9	38.6±2.0	0.0012	0.0510	0.0010	0.3969
Fibrinogen (mg/dL)	342.9±21.0	267.5±9.9	263.4±15.6	269.4±12.7	0.0002	0.0045	0.0007	0.9770
Factor II (%)	92.7±3.3	66.6±2.3	67.8±4.2	66.0±2.8	<0.0001	<0.0001	<0.0001	0.6377
Factor VII (%)	84.8±4.1	49.9±2.4	56.6±4.2	46.9±2.7	<0.0001	<0.0001	<0.0001	0.0839
Factor VIII (%)	120.2±6.5	90.5±4.2	96.3±8.2	87.9±4.8	0.0001	0.0164	<0.0001	0.3238
Factor IX (%)	100.1±3.3	59.9±2.5	65.3±4.5	57.5±2.9	<0.0001	<0.0001	<0.0001	0.1375
Factor X (%)	88.8±3.7	63.8±3.7	59.1±7.4	65.91±4.3	<0.0001	0.0001	0.0001	0.4842
Protein C (%)	100.1±2.2	65.6±2.9	74.5±5.1	61.6±3.3	<0.0001	<0.0001	<0.0001	0.0124
Antithrombin IIa (%)	112.0±3.4	92.8±3.5	96.0±8.8	91.3±3.3	<0.0001	0.0319	0.0007	0.4560

Values are expressed as mean±Standard Error (SE). Unpaired Student's *t* test or one-way ANOVA followed by Fisher's PLSD post test.

**Figure 1:**
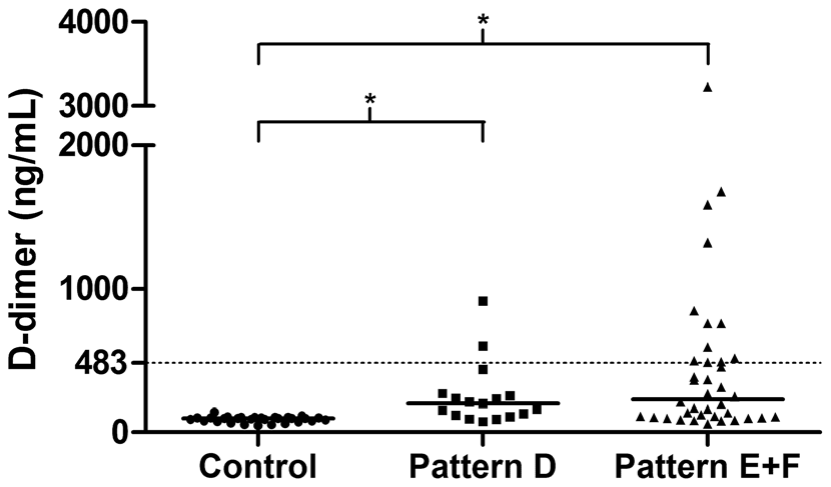
Plasma D-dimer levels in healthy control subjects (Control) and hepatosplenic schistosomiasis patients with either central fibrosis (Pattern D) or advanced periportal cirrhosis (Pattern E+F). The solid horizontal lines show D-dimer median values (96, 199 and 229 ng/mL), while the dashed line indicates the cut-off value for hyperfibrinolysis [14]. *p<0.001; Kruskal-Wallis test.

The levels of D-dimers were significantly higher in the total patient group compared to controls (210 ng/mL [61–3,224 ng/mL] vs. 96 ng/mL [47–190 ng/mL]; median [range]; p<0.001) and as shown in Figure 1 higher levels were a common feature in the patients with advanced periportal fibrosis (229 ng/mL [60.7–3,224 ng/mL]). Using the cut-off value of 483 ng/mL for D-dimer as a measure of hyperfibrinolysis [14], we found that 11 of 38 patients (29%) with advanced periportal fibrosis (Pattern E+F) and a history of upper digestive bleeding exhibited D-dimer levels above this value.

PAI-1 levels were decreased in the patients compared to the control group (65 ng/mL [5–162] vs. 202 ng/mL [17–448 ng/mL]; median [range]; p<0.001), but there were no significant differences (p>0.05) in plasma levels of t-PA and TAFI (data not shown).

All controls and patients were negative for elimination of Schistosoma mansoni eggs in the stool, and also for hepatitis B and C virus markers.

## Discussion

Periportal fibrosis is the main liver consequence of severe infection by *S. mansoni*. It plays a key role in the genesis of portal hypertension and in distorting hepatic parenchyma, which can cause hepatic dysfunction when the fibrosis is extensive. These effects may persist in some patients even after treatment and cure of the infection [7]. Moreover, bleeding episodes can allow progression to decompensated liver disease due to areas of hepatic necrosis caused by hypotension and loss of blood. In our study, we evaluated different patterns of advanced periportal fibrosis in patients with compensated hepatosplenic schistosomiasis, but without ascites, jaundice or hepatic encephalopathy. Pattern E [12] was the most prevalent, and 45% had a previous history of gastrointestinal bleeding. Consistent with our findings reported here, Correia et al [5] observed a high frequency (82%) of thrombocytopenia in patients with hepatosplenic schistosomiasis while other studies have demonstrated progressive deterioration of hepatic function in advanced stages of schistosomasis disease [9]–[11].

Although we report that central fibrosis (Pattern D) is already associated with liver damage, our findings also suggest that γ-GT levels are the best marker of hepatic fibrosis progression. This supports the use of γ-GT as one of three biological markers proposed by Camacho-Lobato and Borges [9] to evaluate the progression of liver dysfunction in schistosomiasis, while Köpke-Aguiar et al. [15] also found γ-GT to be a sensitive indicator along with the platelet count and INR to differentiate patients with or without portal hypertension. Moreover, elevated γ-GT along with ALP was also a feature in hepatosplenic patients with anicteric cholangiopathy, who have alterations in biliary ducts (ductopenia intermediary and small caliber branches) reflecting advanced fibrosis [16].

The liver play a major role in the control of hemostasis, and disturbed liver parenchymal cell function affects the hemostastic system. Such studies report a frequent thrombocytopenia associated with the splenomegaly and portal hypertension [17]. The thrombocytopenia in hepatosplenic schistosomiasis is compensated, at least in part, by increased levels of von Willebrand factor that enable platelets to adhere and aggregate at sites of vascular injury [5], [18], [19]. Köpke-Aguiar et al. [20] also reported that levels of thrombopoetin and reticulated platelets are normal in schistosomiasis patients with portal hypertension and that the bone marrow produces normal amounts of platelets. Thrombocytopenia in schistosomiasis patients may occur because of splenic retention due to poor portal blood drainage, or because platelets are trapped in the sinusoidal spaces of the fibrotic liver [20]. Our study confirms that thrombocytopenia is common in patients with hepatosplenic schistosomiasis, and that this tends to be higher in the advanced stages of periportal fibrosis.

A number of studies have demonstrated reduced vitamin K-dependent coagulation factors in patients with hepatosplenic schistosomiasis [9]–[11] and our findings agree with these reports. Several mechanisms may explain the substantial reductions in coagulation factors, including reduced hepatic synthesis and increased consumption. Impaired carboxylation of precursor molecules is also proposed for factors II, VII, IX and X [11], due to premature release of the protein from damaged hepatocytes, or because of vitamin K-dependent carboxylase deficiency and production of abnormal proteins [10], [11]. Moreover, Tripodi et al. [21] recently highlighted the occurrence of concomitant decreases of both procoagulant and anticoagulant factors in chronic liver disease, mainly in cirrhotic patients. These features escaped attention for many years [11], [21].

Levels of protein C and antithrombin were significantly lower in our patients compared to the healthy controls, presumably reflecting hepatic dysfunction caused by portal hypertension and advanced periportal fibrosis [10], [20]–[22]. Although bleeding events in hepatosplenic schistosomiasis are associated with portal hypertension [20], the deficient production of coagulation factors does not seem to aggravate the situation due to a balance between the reductions in pro- and anti-coagulation proteins. Our results also show that in almost all cases the changes in blood coagulation proteins were greater in patients with advanced periportal fibrosis (Pattern E+F) than those with central fibrosis (Pattern D), although statistical significance for the difference was only reached for protein C. Nevertheless, our data suggest that platelet counts, Factor VII and protein C are good predictors of advanced fibrosis.

Fibrinolysis was studied in patients with decompensated hepatosplenic schistosomiasis by El-Bassiouni et al. [23] who reported high concentrations of t-PA and low levels of PAI-1. Although PAI-1 was also decreased in our patients, the level of t-PA (and TAFI) did not differ between our patients and controls. In addition, we found increased levels of D-dimer, most notably in those patients with advanced periportal fibrosis (Pattern E+F). Plasma D-dimer concentration reflects the degree of thrombin turnover and consequently increased levels are a good marker of recent coagulation and fibrinolysis. Indeed, Primignani et al. [14] suggested D-dimer as a predictor of death in patients with liver cirrhosis. Using the cut-off value proposed by Primignani et al. [14], one-third of our patients with Pattern E+F were considered to have hyperfibrinolysis, which may increase their risk of a bleeding event. Therefore, we recommend for patients eligible for surgical procedures that D-dimer be measured during the pre-surgical evaluation, in addition to PT and platelet count, to better assess the risk of bleeding.

One limitation of our study is that it was conducted at a single hospital, the Hospital das Clinicas, UFPE. This is the reference hospital for schistosomiasis in Pernambuco State and receives the most severe cases of schistosomiasis, usually patients with a history of one or more episodes of gastrointestinal bleeding and hence a high proportion with abnormal liver function tests. Thus, the findings from our study may not extrapolate to all patients from endemic areas who present with the hepatosplenic form of the disease.

In conclusion, our study verified that coagulation abnormalities in hepatosplenic schistosomiasis are due to liver fibrosis and portal hypertension, and additionally demonstrated that these abnormalities increase in advanced periportal fibrosis and that reduced levels of protein C may be a good marker of hepatic fibrosis progression.

## Supporting Information

Checklist S1 STROBE checklist.
(DOC)
